# Evaluating the effectiveness of stain normalization techniques in automated grading of invasive ductal carcinoma histopathological images

**DOI:** 10.1038/s41598-023-46619-6

**Published:** 2023-11-22

**Authors:** Wingates Voon, Yan Chai Hum, Yee Kai Tee, Wun-She Yap, Humaira Nisar, Hamam Mokayed, Neha Gupta, Khin Wee Lai

**Affiliations:** 1 Department of Mechatronics and Biomedical Engineering, Faculty of Engineering and Science, Lee Kong Chian, Universiti Tunku Abdul Rahman, Kampar, Malaysia; 2 Department of Electrical and Electronic Engineering, Faculty of Engineering and Science, Lee Kong Chian, Universiti Tunku Abdul Rahman, Kampar, Malaysia; 3 Department of Electronic Engineering, Faculty of Engineering and Green Technology, Universiti Tunku Abdul Rahman, 31900 Kampar, Malaysia; 4 Department of Computer Science, Electrical and Space Engineering, Lulea University of Technology, Lulea, Sweden; 5 School of Electronics Engineering, Vellore Institute of Technology, Amaravati, AP India; 6 Department of Biomedical Engineering, Universiti Malaya, 50603 Kuala Lumpur, Malaysia

**Keywords:** Biomedical engineering, Diagnostic markers

## Abstract

Debates persist regarding the impact of Stain Normalization (SN) on recent breast cancer histopathological studies. While some studies propose no influence on classification outcomes, others argue for improvement. This study aims to assess the efficacy of SN in breast cancer histopathological classification, specifically focusing on Invasive Ductal Carcinoma (IDC) grading using Convolutional Neural Networks (CNNs). The null hypothesis asserts that SN has no effect on the accuracy of CNN-based IDC grading, while the alternative hypothesis suggests the contrary. We evaluated six SN techniques, with five templates selected as target images for the conventional SN techniques. We also utilized seven ImageNet pre-trained CNNs for IDC grading. The performance of models trained with and without SN was compared to discern the influence of SN on classification outcomes. The analysis unveiled a *p*-value of 0.11, indicating no statistically significant difference in Balanced Accuracy Scores between models trained with StainGAN-normalized images, achieving a score of 0.9196 (the best-performing SN technique), and models trained with non-normalized images, which scored 0.9308. As a result, we did not reject the null hypothesis, indicating that we found no evidence to support a significant discrepancy in effectiveness between stain-normalized and non-normalized datasets for IDC grading tasks. This study demonstrates that SN has a limited impact on IDC grading, challenging the assumption of performance enhancement through SN.

## Introduction

Invasive ductal carcinoma (IDC) is widely recognized as the most common form of breast cancer, accounting for over 80% of breast cancer cases^1^. IDC grading is a crucial factor in determining the prognosis of IDC and plays a critical role in evaluating its clinical outcome. Henson et al.^2^ found that the accuracy of IDC diagnosis improved when both the IDC grade and lymph node condition were considered. Similarly, the research conducted by Frkovic-Grazio and Bracko^3^ demonstrated that IDC grading effectively predicts the behavior of the tumor, particularly for early-stage, small tumors. Schwartz et al.^4^ also uncovered that when undergoing mastectomy, patients with high-grade IDC faced higher fatality rates and more frequent axillary lymph node involvement compared to those with lower-grade IDC. These findings highlight the significance of IDC grading in the prognostic evaluation of IDC.

The standard method of grading IDC is the Nottingham Grading Scheme (NGS), which is a semi-quantitative system based on three morphological features of IDC: mitotic count, nuclear pleomorphism, and degree of tubule formation^5^. These three criteria result in a total score that can be divided into Grades 1 to 3, which indicate the aggressiveness of the tumor. Lower-grade IDC is less aggressive, while higher-grade IDC is more aggressive^6^. Although manual IDC grading is still the standard, it can be time-consuming^7^ and prone to high intra- and inter-observer variations, with agreement among pathologists reaching only 75.3% at best^8^. To address these limitations, automated IDC grading systems, a type of computer-aided diagnostic (CAD) technique, have been developed^9^.

The development of automated IDC grading systems has significantly advanced from traditional handcrafted feature extraction methods^10–14^ to the application of deep learning techniques^15–20^. This evolution extends beyond IDC grading, as deep learning also finds widespread utilization in various histopathological applications^21,22^. The process of generating digital IDC histopathological images involves several steps including the collection of IDC tissues, formalin fixation, paraffin section embedment, and staining with hematoxylin and eosin (H&E)^23,24^. The slides are then digitized using Whole Slide Imaging technology^25^. H&E staining, the standard protocol in histopathological studies, highlights cell nuclei in blue and different components such as cytoplasm and connective tissue with various shades of pink^26^.

There is controversy surrounding the impact of Stain Normalization (SN) in recent breast cancer histopathological studies. Some studies have indicated that SN has no effect on classification results^26–30^, while others have claimed that SN improves classification outcomes^31–35^. The purpose of SN is to address color inconsistencies in digital H&E-stained images caused by external factors such as the temperature of staining solutions, fixation characteristics, imaging device characteristics^36,37^, and variations in light sources, detectors, or optics during slide digitization^38^. SN normalizes the color values of source images by matching the overall color distribution of target images^39^. However, the effectiveness of SN in improving classification results is uncertain due to the conflicting results in the literature.

In light of this, our aim is to investigate the effectiveness of SN in the breast cancer histopathological classification task using convolutional neural networks (CNNs), with a specific focus on classifying the Four Breast Cancer Grades (FBCG)^19^ dataset into four IDC grades. We attempted to answer the question: "Is SN effective in the IDC grading task?" by conducting a statistical significance analysis using Student's *t*-test with the significance level, *α* = 0.05. Below are our statements of null and alternative hypotheses:

1. Null hypothesis ***H***_***0***_: A CNN trained with a stain-normalized dataset has no effect on the IDC grading accuracy.
2. Alternative hypothesis ***H***_***1***_: A CNN trained with a stain-normalized dataset has an effect on the IDC grading accuracy.

In this paper, we selected six types of conventional and deep learning-based SN techniques to study their effectiveness with the IDC grading FBCG dataset using CNNs. The conventional methods, including Reinhard^40^, Macenko^41^, Structure-preserving Color Normalization (SPCN)^42^ and Adaptive Color Deconvolution (ACD)^43^ techniques, require a template as the stain target reference to stain-normalize the images. Hence, we selected five templates from the Patch Camelyon (PCam) dataset^44^ (a dataset derived from Camelyon16^45^) for the conventional methods. For the deep learning-based SN methods, we utilized the Camelyon16^45^ pre-trained StainGAN^46^ and StainNet^47^ to stain-normalize the images in the FBCG dataset. After normalizing the images, we implemented seven pre-trained CNNs: (1) EfficientNet-B0^48^, (2) EfficientNet-V2-B0^49^, (3) EfficientNet-V2-B0-21k^49^, (4) ResNet-V1-50^50^, (5) ResNet-V2-50^51^, (6) MobileNet-V1^52^, and (7) MobileNet-V2^53^ as feature extractors in our IDC grading models to conduct the classification task. Our source code can be accessed publicly from: https://github.com/wingatesv/SN_IDC_Grading.

In this study, we have made the following contributions and reached the following conclusions:

1. We conducted a comprehensive evaluation of six conventional and deep learning-based SN techniques on the task of IDC grading using the FBCG dataset.
2. We conducted a systematic review of ten recent studies that investigated the efficacy of SN in breast cancer histopathological classification. The findings are presented in the section on related works.
3. Our results suggest that if SN is deemed necessary in the image pre-processing pipeline, StainGAN, StainNet, and ACD techniques are preferable to Reinhard, Macenko, and SPCN techniques.
4. Our statistical analysis revealed a *p*-value of 0.11 when comparing the mean balanced accuracy scores between models trained with the StainGAN-normalized FBCG dataset (the best performing SN technique), which achieved a score of 0.9196, and those trained with the non-normalized dataset, which scored 0.9308. This implies that we found no evidence of a significant difference in effectiveness between stain-normalized and non-normalized datasets for grading tasks.
5. Our findings challenge the assumption that stain normalization significantly improves histopathological classification tasks, as we found no evidence of a significant discrepancy in effectiveness between stain-normalized and non-normalized datasets for IDC grading tasks.

Our study provides insights into the effectiveness of SN techniques in breast cancer histopathological studies, with a particular focus on the IDC grading task. While there has been some debate over the impact of SN on classification outcomes, our research has shown that models trained with the non-normalized dataset can be just as effective as those trained with StainGAN-normalized images. Our findings provide a valuable contribution to the field and can help guide future research on SN techniques. We are optimistic that our study will encourage researchers to approach the topic with a critical lens and produce even more promising results in the future.

## Related works

In this section, we examine the development of automated IDC grading systems and various SN methods. The SN techniques are divided into two categories: (1) conventional approaches and (2) deep learning-based approaches. Next, we present studies that investigated the effect of SN in various breast cancer histopathological image classification tasks.

### Automated IDC grading systems

The development of automated IDC grading systems has progressed from manual feature extraction methods to deep learning-based approaches. For instance, Doyle et al.^11^ proposed a method for extracting textural and architectural features by using spectral clustering to reduce the dimensionality of the extracted features, which were then used to classify the IDC grades with a support vector machine. Basavanhally et al.^13^ employed a multifield-of-view (multi-FOV) classifier to identify the most salient image features from multiple FOV of varying sizes for the purpose of IDC grading. Dimitropoulos et al.^14^ transformed images into vectors of locally aggregated descriptors (VLAD) representations based on the Grassmann manifold. They then calculated the VLAD encoding of each image on the manifold to determine the IDC grade. However, these methods are heavily reliant on features and are computationally intensive, with a lack of heuristics for feature extraction^18^. As a result, more recent studies have shifted towards deep learning methods, specifically Convolutional Neural Networks (CNNs)^15,17,18,54^. For example, Senousy et al.^18^ developed an entropy-based elastic ensemble of CNNs (3E-Net) for IDC grading, and Yan et al.^55^ created a nuclei-guided network (NGNet) with a nuclei-guided attention module for IDC grading as well. In terms of transfer learning, Zavareh et al.^20^ used the VGG16 model as a feature extractor in the BCNet to grade IDC. Similarly, Voon et al.^56^ evaluated the performance of seven pre-trained CNN models in the IDC grading task. In this study, we adopted the model implementation of Voon et al.^56^ which utilized transfer learning. This approach was chosen due to the improved performance of CNNs when trained on a limited number of training images.

### Stain normalization methods

#### Conventional stain normalization methods

Conventional approaches to Stain Normalization (SN) in histopathological images typically involve the analysis, transformation, and alignment of the color components of images^47^. The Reinhard method^40^ normalizes the images by adjusting the statistical color distribution of the source image to match that of a template image while preserving the background color and color intensities. The Macenko technique^41^ employs Single Value Decomposition (SVD) to form a plane that projects information, determining the corresponding angles and finally estimating the color matrix. The Khan method^39^ identifies the stain color of the source image using the Stain Color Descriptor (SCD), then uses a Relevance Vector Machine (RVM) to determine the position of each stain and transfers the color from the template to the source image using a non-linear spline-based color normalization technique. The Structure-Preserving Color Normalization (SPCN)^42^ decomposes the source images into sparse stain density maps, combining the stain of the template image to change only the color while preserving the structures. The Adaptive Color Deconvolution (ACD)^43^ normalizes the stains by integrating optimization to approximate the parameters of stain separation and color normalization. This technique, based on Color Deconvolution (CD)^57^, optimizes the estimation of stain parameters. However, these methods^39–43^ depend on a reference image to approximate the stain parameters, presenting a challenge to encompass all staining patterns or represent all input images. As a result, the use of suboptimal reference images may lead to incorrect estimation of stain parameters and result in inaccurate outcomes^58,59^.

#### Deep learning-based stain normalization methods

Recently, a significant shift has been observed towards the adoption of deep learning-based techniques for stain normalization (SN). This approach offers a departure from traditional methods that rely on template images^46,47,60,61^. Zanjani et al.^60^ proposed the use of generative adversarial networks (GANs) to learn the relationship between image content structures and their respective color attributes, thereby facilitating color alignment without relying on statistical properties. Shaban et al.^46^ extended this work by developing StainGAN, a CycleGAN-based technique^62^ that enables the transfer of stain style from one domain to another without the need for paired data. Similarly, Kang et al.^47^ introduced StainNet, a method that leverages the output of StainGAN to better understand the pixel-wise color mapping relationship within a given dataset. In our current study, we sought to investigate the effectiveness of SN in the context of IDC grading. To this end, we considered a diverse range of techniques, including Reinhard, Macenko, Structure-Preserving Color Normalization (SPCN), Adaptive Color Deconvolution (ACD), StainGAN, and StainNet.

### Study of stain normalization in breast cancer histopathological images classification

This section presents an overview of the prior studies that have compared the performance of models trained with stain-normalized and non-normalized inputs in the context of breast cancer histopathological image classification. Despite the numerous studies in this field, there is still considerable controversy regarding the efficacy of SN on the performance of these models^26–35^.

On one hand, several studies^26–30^ have reported that SN has no significant impact on the performance of the models. For example, Gupta et al.^27^ evaluated the classification performance of different texture descriptors and contemporary classifiers using Reinhard-normalized BreaKHis^63^ dataset and found that SN did not lead to improvement in the results. Similarly, Tellez et al.^26^ compared the performance of CNNs trained on Camelyon17^64^ dataset using Macenko and Berjnodi^36^ SN techniques, and revealed that SN did not enhance the performance, with the CNN trained on the non-normalized dataset even outperforming those trained on the stain-normalized datasets. These findings were supported by Kumar et al.^28^, who found that a pre-trained VGG16 model trained on the non-normalized BreaKHis dataset outperformed the identical model trained on the Macenko-normalized dataset. Hameed et al.^29^ also found that the performance of deep learning-based ensemble models declined when using stain-normalized datasets, while Hameed et al.^30^ failed to find any performance improvement when the pre-trained Xception model was trained on the Colsanitas dataset^29^ with Reinhard, Macenko, CD, and SPCN SN techniques.

On the other hand, several studies^31–35^ have suggested that SN does indeed improve the performance of the models. For example, Nawaz et al.^31^ fine-tuned the AlexNet model on the ICIAR2018 dataset^65^ and found that the AlexNet trained on the Macenko-normalized dataset outperformed the model trained on the non-normalized dataset. Shahidi et al.^35^ compared the performance of different CNNs on Macenko-normalized and non-normalized BreaKHis datasets and found that SN improved the model performance. Munien and Viriri^32^ implemented seven pre-trained EfficientNets to classify the original, Reinhard-normalized, and Macenko-normalized ICIAR2018 datasets. The results showed that models trained with stain-normalized datasets outperformed models trained with the non-normalized dataset. Salvi et al.^33^ attempted to classify the BACH challenge^65^ dataset with Stain Color Adaptive Normalization (SCAN) technique^66^. The authors found that the normalized dataset obtained better results than the non-normalized dataset. Similarly, Alkassar et al.^34^ utilized an ensemble of models to classify Khan-normalized and non-normalized BreaKHis datasets. The results showed that the models trained with the Khan-normalized dataset outperformed those trained with the non-normalized dataset. Therefore, we can conclude that these studies highlighted the benefits of SN in the classification task.

These inconsistent findings have created a knowledge gap in the application of SN in IDC grading, leading to confusion among researchers about the effectiveness of SN in future studies. In light of this, we set out to answer the question: "Is SN effective in the IDC grading task?" by investigating the effectiveness of six conventional and deep learning-based SN techniques on the IDC grading task using the FBCG dataset and CNNs.

## Methodology

### Overview

**Figure 1:**
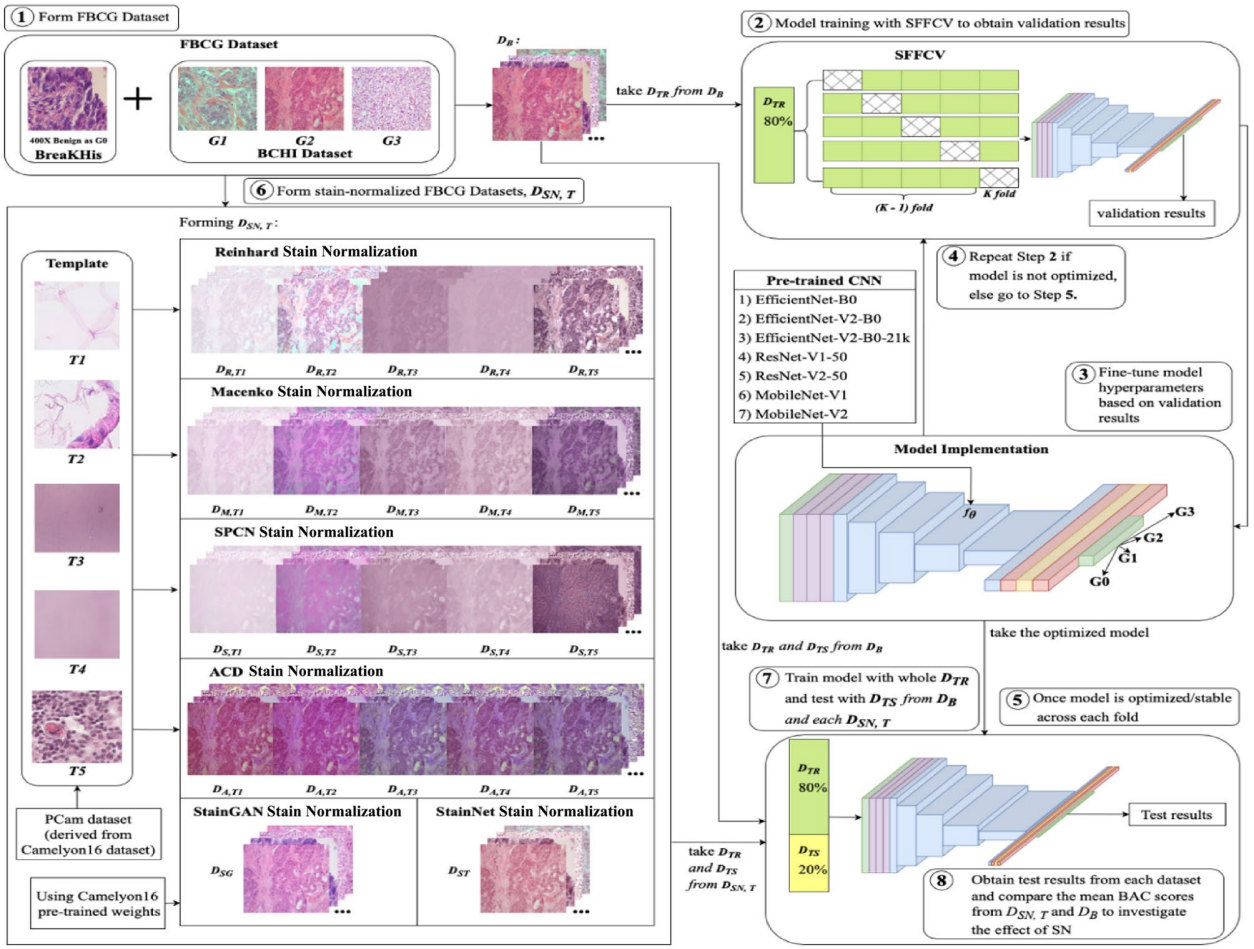
The overall methodology of the study. (1) The FBCG dataset is assembled by combining images from the 400X Benign class of the BreaKHis dataset and images from the BCHI dataset. (2) To evaluate model stability, the implemented model is trained with* D*_*TR*_ from* D*_*B*_ using the Stratified Five-fold Cross-validation (SFFCV). (3) The hyperparameters of the model are optimized until the model is stable across each fold. (4) The SFFCV process is repeated until the model is optimized. (5) Once satisfactory model performance is achieved, (6) the FBCG datasets undergo stain normalization using various techniques to form* D*_*SN, T*_. (7) Lastly, each* D*_*SN, T*_ and* D*_*B*_ is fed forward into the model to retrain, followed by (8) obtaining the final test results.

In this section, we provide an outline of the six SN techniques used in the IDC grading task. We also elucidate the implementation details, which include aspects such as the FBCG dataset, image pre-processing procedures, CNN model implementations, and the evaluation metric. All experimentations were conducted using Python and TensorFlow Keras on the Google Collaboratory platform. The technical specifications for these experiments included a 2.30 GHz Intel® Xeon® CPU, up to 32 GB RAM, and an NVIDIA P100 or T4 GPU. We ensure that all procedures adhered to relevant guidelines and regulations. Figure 1 illustrates the general methodology of the study.

### Stain normalization

SN aims to normalize the color values of the source images by aligning the overall color distribution with that of target images. Our study explored six types of SN techniques, specifically Reinhard^40^, Macenko^41^, SPCN^42^, ACD^43^, StainGAN^46^ and StainNet^47^ (Note that the employed StainGAN and StainNet were pre-trained on the Camelyon16 dataset^45^).

#### Template selection

**Figure 2:**
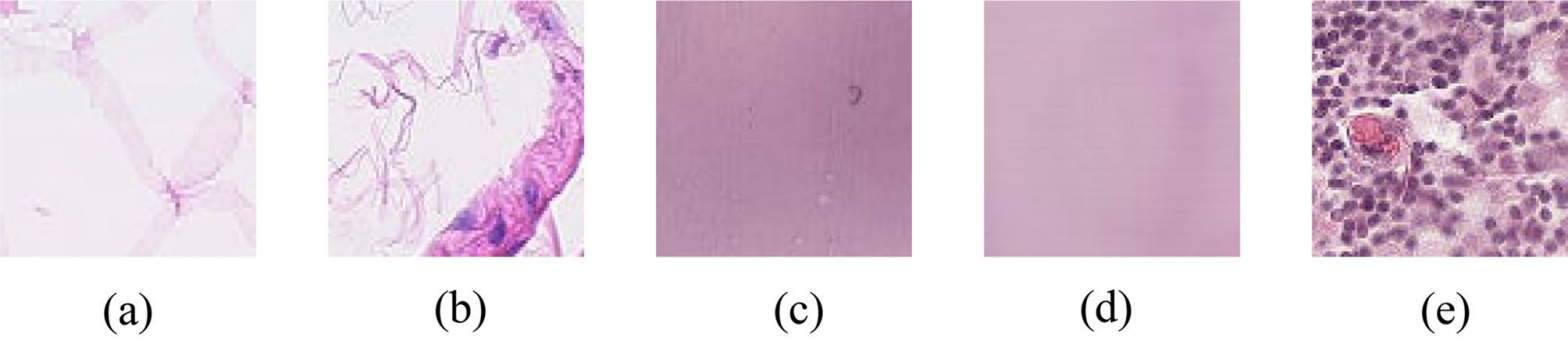
Five templates selected from PCam train set: (**a**) *T*1, (**b**) *T*2, (**c**) *T*3, (**d**) *T*4 and (**e**) *T*5.

The selection of an appropriate template is crucial for conventional SN techniques, which rely on a single template to perform color conversion between source and target images. If the template is not chosen wisely, the performance of SN techniques may be compromised^47^. Therefore, we selected five templates where $T \in \{T1, T2, T3, T4, T5\}$ (see Fig. 2) from the PatchCamelyon (PCam) dataset^44^, our target dataset, to investigate the impact of each template on the SN techniques. It is imperative to note that the selection of these templates was not selected based on subjective decisions. Instead, they were chosen based on a methodical process that involved generating an average image from the target dataset and using similarity functions to compare this average image with image samples within the target dataset. This approach helped us identify a template that most accurately reflects the overall color staining distribution of the dataset.

##### Average image generation

Before selecting any template, we generated an image ${I}_{avg}$ that represents the average pixel values of the target dataset. In this case, we selected the PCam train set as the target dataset ${D}_{t}$ to ensure a fairer comparison with the StainGAN and StainNet SN techniques. PCam is a public histological dataset which comprises patch-wise images with dimensions of 96 by 96 pixels. These images are extracted from histological scans of lymph node sections from the Camelyon16 Challenge, which focuses on breast cancer metastasis. To generate ${I}_{avg}$, all 262,144 images from the PCam train set were converted into floating-point arrays, followed by summing up the arrays to yield the average pixel values.

##### Templates 1 and 2

Template 1 $$(T1)$$ and Template 2 ($$T2)$$ were selected using cosine similarity $${SIM}_{C}$$. This method computes the dot product of two vectors and divides it by the product of their magnitude to determine their similarity. Specifically, we computed the $${SIM}_{C}$$ between $${I}_{avg}$$ and image $$X \in {D}_{t}$$ to locate *X* that most resembles $${I}_{avg}$$, resulting in $$T1$$. Likewise, selecting $$T2$$ adopted a similar approach. However, the most dominant color, *C*_*dom*_ of $${I}_{avg}$$ and image $$X \in {D}_{t}$$ were obtained, followed by forming image $${I}_{avg, dom}$$ and $${I}_{Dt, dom}$$ based on each dominant color, respectively. Subsequently, we computed the $${SIM}_{C}$$ between $${I}_{avg, dom}$$ and $${I}_{Dt, dom}$$, resulting in $$T2$$. Equation (1) formally describes the $${SIM}_{C}$$:

1 $${SIM}_{C}\left(A, B\right)=\frac{\sum_{i=1}^{n}{A}_{i}{B}_{i}}{\sqrt{\sum_{i=1}^{n}{A}_{i}^{2}}\sqrt{\sum_{i=1}^{n}{B}_{i}^{2}}}$$

where *A* and *B* denote vectors with *n-th* number of pixels flatten from ${I}_{avg}$ and image $X \in {D}_{t}$ or ${I}_{avg, dom}$ and ${I}_{Dt, dom}$. Equation (2) formally describes the *C*_*dom*_*:*

2 $${C}_{dom}=\mathrm{arg}\underset{c \in C(P)}{\mathrm{max}}N(c)$$

where *P* denotes the set of all pixels in an image, *C(p)* denotes the function that returns the color of pixel *p*, and *N(c)* denotes the function that returns the number of pixels of color *c* in the image.

##### Templates 3, 4 and 5

For Templates 3, 4, 5, we used different selection methods. Template 3 $$(T3)$$ was selected using the Mean Square Error $$MSE$$, while Template 4 ($$T4)$$ was chosen based on the Structural Similarity Index $$SSIM$$. Similar to $$T1$$ and $$T2$$, we computed the $$MSE$$ and *SSIM* between $${I}_{avg}$$ and image $$X \in {D}_{t}$$ to find the most similar *X*, resulting in $$T3$$ and $$T4$$. For Template 5 $$(T5)$$, we identified the most dominant color in $${I}_{avg}$$ and image $$X \in {D}_{t}$$, We then formed images $${I}_{avg, dom}$$ and $${I}_{Dt, dom}$$, based on each dominant color. Then, we computed the $$MSE$$ or *SSIM* between $${I}_{avg, dom}$$ and $${I}_{Dt, dom}$$, resulting in $$T5$$ (note that the results of $$MSE$$ and $$SSIM$$ are identical). Equations (3) and (4) describe $$MSE$$ and $$SSIM$$ respectively as followed:

3 $$MSE\left({I}_{A}, {I}_{B}\right)=\frac{1}{n}\sum_{i=1}^{n}{({I}_{A, i}-{I}_{B,i})}^{2}$$

4 $$SSIM\left({I}_{A}, {I}_{B}\right)=\frac{(2{\mu }_{IA}{\mu }_{IB}+{C}_{1})(2{\sigma }_{IAIB}+{C}_{2})}{({\mu }_{IA}^{2}+{\mu }_{IB}^{2}+{C}_{1})({\sigma }_{IA}^{2}+{\sigma }_{IB}^{2}+{C}_{2})}$$

where ${I}_{A}$ and ${I}_{B}$ denote input and output image matrices with *n-th* number of pixels respectively, ${\mu }_{IA}$ and ${\mu }_{IB}$ denote the luminance of ${I}_{A}$ and ${I}_{B}$ respectively, ${\sigma }_{IA}$ and ${\sigma }_{IB}$ denote the contrast of ${I}_{A}$ and ${I}_{B}$ respectively, ${C}_{1}$ and ${C}_{2}$ denote constants to ensure stability where ${C}_{1}$ and ${C}_{2} >0$.

#### Reinhard stain normalization technique

The Reinhard SN technique normalizes the source image ${I}_{s}$ by aligning the mean $\mu$ and standard deviation $\sigma$ with a template *T*. Algorithm *1* outlines the workflow of the Reinhard algorithm. The Reinhard method transforms the *RGB* images to $l\alpha \beta$ color space where *l* represents the achromatic channel, *α* denotes the chromatic blue-yellow channel and *β* signifies the chromatic green–red channel. Subsequently, the following Eqs. (5), (6) and (7) are applied to perform the Reinhard transformation, then convert the output image ${I}_{out}$ back to *RGB* color space^40,68^.

5 $${l}_{2}=\mu \left({l}_{1}\right)+\left(l-\mu \left(l\right)\right)\odot (\sigma \left({l}_{1}\right) \oslash \sigma \left(l\right))$$

6 $${\alpha }_{2}=\mu \left({\alpha }_{1}\right)+\left(\alpha -\mu \left(\alpha \right)\right) \odot (\sigma \left({\alpha }_{1}\right) \oslash \left(\alpha \right))$$

7 $${\beta }_{2}=\mu \left({\alpha }_{1}\right)+\left(\beta -\mu \left(\beta \right)\right) \odot (\sigma \left({\beta }_{1}\right) \oslash \sigma \left(\beta \right))$$

where $l, {l}_{1} \,\text{and} \,{l}_{2}$ depict the ${I}_{S}, T \,\text{and} \,{I}_{out}$ in the *l* space respectively; $\alpha , {\alpha }_{1} \,\text{and}\, {\alpha }_{2}$ depict the ${I}_{S}, T \,\text{and}\, {I}_{out}$ in the $\alpha$ space respectively; $\beta , { \beta }_{1}\mathrm\,\text{and}\,{\beta }_{2}$ depict the ${I}_{S}, T \,\text{and}\, {I}_{out}$ in the $\beta$ space respectively; $\odot$ denotes element-wise multiplication and $\oslash$ denotes element-wise division.

**Algorithm 1:**
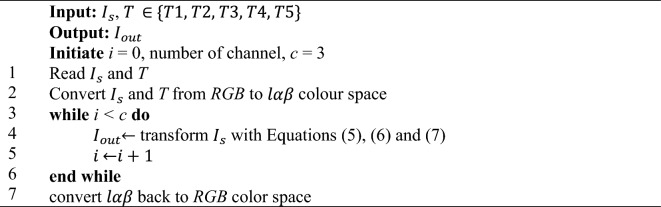
Reinhard Technique

#### Macenko stain normalization technique

The Macenko technique separates stains by identifying the fringe of pixel distribution in the Optical Density space (*OD*). Algorithm *2* provides a detailed description of the Macenko algorithm. Similar to Reinhard, Macenko converts the *RBG* image to *lαβ* color space, followed by transforming the colors into *OD* values with Eq. (8):

8 $$OD= {-log}_{10}({I}_{s})$$

The color transformation to *OD* values provides a space where a linear stain fusion yields a linear fusion of *OD* values. Subsequently, the transparent pixels are removed if the *OD* value is below a specific threshold. The *OD* value is split into two matrices, given by Eqs. (9) and (10).

9 $$OD=V*S$$

10 $$S={V}{\prime}*OD$$

where *S* represents each stain saturation and *V* denotes stain vector matrix. Equations (8) and (9) locate the stain vector of each image based on the color (if *OD* = 0, then the corresponding pixel = white; the stain is absent). Next, we compute the singular value decomposition (SVD) on the *OD* value, followed by locating the stain vector terminal points using the Geodesic path^37^. We can then assess the plane, which is created by vectors. The procedure is conducted by creating a plane with two vectors corresponding to the two most significant *SVD* values. Afterwards, we project all *OD* values into the plane, normalizing to unit length and curving the projected line. With these, we can compute each angle to the first *SVD* direction, thus, mapping the direction in the plane. As a result, the pixel intensity histogram can be computed, followed by determining the concentration of each stain with the H&E matrix in relation to the *OD* values. Finally, we can yield ${I}_{out}$ by using the H&E matrix with the normalized stain concentration^41,68^.

**Algorithm 2:**
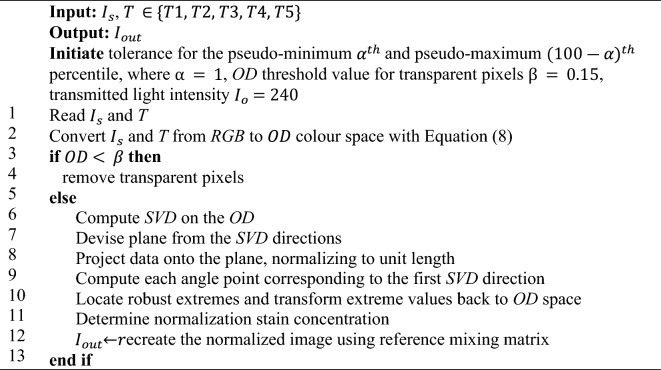
Macenko Technique

#### Structure-preserving color normalization

Structure-Preserving Color Normalization (SPCN)^42^ operates by decomposing ${I}_{s}$ into sparse stain density maps while integrating the stain from *T*. Algorithm *3* illustrates the implementation of SPCN. Given $I\in {\mathbb{R}}^{m\times n}$ is the *RGB* image matrix, where *m* denotes the number of *RGB* channels and *n* denotes the number of pixels. Let $w\in {\mathbb{R}}^{m\times r}$ be the stain matrix with columns representing the chromatic variance of each stain, where *r* represents the stain number. Let $H\in {\mathbb{R}}^{r\times n}$ represents the stain density maps where the rows denote the stain concentration. Thus, *I* is described as:

11 $$I= {I}_{o}{e}^{-WH}$$

Let *V* be the OD maps then,

12 $$V= log(\frac{{I}_{o}}{I})$$

By utilizing Eq. (11), we can form:

13 $$V= WH$$

where *V* = observation matrix, *H* = stain density map matrix, and *W* = stain color appearance matrix. Next, we implement the sparse non-negative matrix factorization (SNMF) for stain separation. Based on the Beer-Lambert law, the *RGB* image is converted into the *OD* maps with Eq. (14). Then, the sparseness constraint is added in Eq. (11). SNMF separates stain with ${l}_{1}$= sparseness and ${H}_{j}$ = stain mixing coefficient where, *j* = index of stains that is $j=\mathrm{1,2}, \dots \dots r,$.

14 $$\varphi \left(p\right)= -\mathrm{log}(V(p))$$

where $\varphi$ denotes as the OD space, *p* = pixel intensity where, $p\in pixel P$.

15 $$min\frac{1}{2}{\Vert V-WH\Vert }_{F}^{2}+\lambda {\sum }_{j=1}^{r}{\Vert H(j,:)\Vert }_{1}, W, H\ge 0$$

16 $${\Vert W(:,j)\Vert }_{2}^{2}=1$$

where $\lambda$ = the sparsity and regularization parameter. Additional constraints on *W* and *H* will decrease the solution space of $W/\alpha$ and $\alpha H$, where $\alpha$ is a positive value. Equation (12) represents a non-convex optimization problem, which can be addressed by alternating optimizing one parameter of *H* and *W* while holding the other constant. Elements are randomly selected from the optical density *V* to initialize the color appearance matrix.

Subsequently, we transfer the color $\mu$ of *T* to ${I}_{s}$ while approximating the color appearance matrix for stain normalization. Utilizing the SNMF, we factorize the stain density maps ${V}_{s}$ into ${W}_{s}{H}_{s}$ and ${V}_{t}$ into ${W}_{t}{H}_{t}$. Afterwards, the stain density maps of source ${H}_{s}$ are merged with the template ${W}_{t}$ color appearance matrix instead of the source color appearance matrix ${W}_{s}$ to produce the normalized image. As a result, stain density map *H* maintains the structure while the color appearance matrix *W* maintains changes in the color appearance. Lastly, the inverse Beer-Lambert transformation (BLT) is applied to the normalized stains to obtain ${I}_{out}$^42,68^.

**Algorithm 3:**
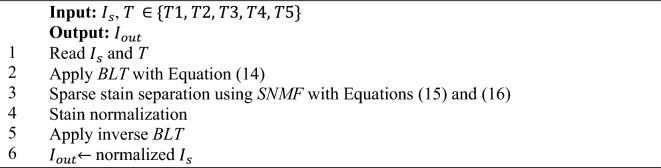
Structure-Preserving Color Normalization

#### Adaptive color deconvolution technique

Adaptive Color Deconvolution (ACD)^43^ normalizes stains by integrating optimization to approximate the stain separation parameters and color normalization. ACD is based on color deconvolution (CD)^57^. Let ${x}_{i}\in {\mathbb{R}}^{3\times 1}$ denote the *RGB* values of each *i-th* pixel in ${I}_{s}$. CD is described with Eqs. (17) and (18):

17 $${o}_{i}= -\mathrm{ln}(\frac{{x}_{i}}{{I}_{max}})$$

18 $${s}_{i}= D\cdot {o}_{i}$$

Where ${o}_{i}\in {\mathbb{R}}^{3\times 1}$ represents the *OD* of *RG*B channels, ${I}_{max}$= background intensity, and $D\in {\mathbb{R}}^{3\times 3}$ = CD matrix. The separated densities of stains are denoted ${s}_{i}= {({h}_{i}, {e}_{i},{d}_{i})}^{T}$, where ${h}_{i}=$ hematoxylin stain, ${e}_{i}$ = eosin stain, and ${d}_{i}$ = separation residual. CD matrix *D* is decided by a Stain Color Appearance (SCA) matrix *M*, where $D= {M}^{-1}$. Therefore, ACD is derived by applying a stain-weight matrix $W= diag({w}_{h}, {w}_{e},1)$ to directly optimize the stain separation parameters and color normalization. We modify Eq. (18) to form Eq. (19):

19 $${s}_{i}=W\cdot D\cdot {o}_{i}$$

The SCA matrix $M=({m}_{h}, {m}_{e}, {m}_{d})$, where ${m}_{j}\in {\mathbb{R}}^{3\times 1} (j=h,e,d)$ is a unit vector representing the contributions of the *j-th* stain to the *RGB* channels intensities. *M* is determined by $\varphi$, representing as M ($\varphi )$ and CD matrix *D* as D ($\varphi )$, where $\varphi$ is a collection of six-degree variables $\varphi =\{{\alpha }_{h}, {\beta }_{h}, {\alpha }_{e}, {\beta }_{e}, {\alpha }_{d}, {\beta }_{d}\}$. Thus, we perform optimization by minimizing the objective function ${\mathcal{L}}_{ACD}$^43^ of variables $\varphi$ and *W*:

20 $$(\widehat{\varphi }, \widehat{W})=\mathrm{argmin}{\mathcal{L}}_{ACD}(\varphi , W)$$

We employed the gradient descent to solve ${\mathcal{L}}_{ACD}(\varphi , W)$ which is continuous and differentiable for variables *φ* and *W*. By resolving ${\mathcal{L}}_{ACD}$, $\widehat{\varphi }$ and $\widehat{W}$ can be obtained, followed by determining the adaptive matrices *M* ($\widehat{\varphi })$ and *D* ($\widehat{\varphi })$ for the ${I}_{s}$. After the optimization, we obtain the adaptive variables for the stain separation $\widehat{D}$ and stain intensity normalization $\widehat{W}$. Subsequently, we separate the ${I}_{s}$ stain components with $\widehat{D}$, followed by weighting with $\widehat{W}$. Lastly, we recombine the weighted stain components with the SCA matrix of the template *T* $\overline{M }$ to obtain ${I}_{out}$*.* The following Eqs. (17), (21) and (22) summarize ACD techniques for the *i-th* pixel ${x}_{i}$:

17 $${o}_{i}= -\mathrm{ln}(\frac{{x}_{i}}{{I}_{max}})$$

21 $$\overline{{o }_{i}}= \overline{M}\cdot \widehat{W}\widehat{D}\cdot {o }_{i}$$

22 $${I}_{out}=\overline{{x }_{i}}= \mathrm{exp}(-\overline{{o }_{i}})\cdot {I}_{max}$$

**Algorithm 4:**
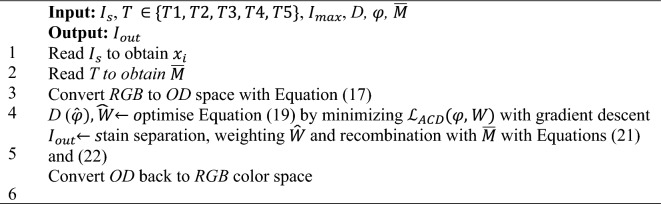
Adaptive Color Deconvolution

#### StainGAN

StainGAN^46^ is inspired by CycleGAN^62^ that transfers stains between two domains without requiring paired data from both domains. StainGAN is composed of two pairs, each consisting of a generator and a discriminator. The first pair (*G*_*A*_ and *D*_*A*_) aims to map images from Domain *B* to Domain *A* ${G}_{A}:{X}_{B}\to {X}_{A}$. The Generator *G*_*A*_ aims to generate images that match Domain A. The discriminator *D*_*A*_ tries to verify if images originate from Domain *A* or the fake generated ones. The other pair (*G*_*B*_ and *D*_*B*_) undergoes the same process in the reverse direction, ${G}_{B}:{X}_{A}\to {X}_{B}$ as:

23 $$\widehat{{X}_{A}}={G}_{A}\left({X}_{B};{\theta }_{A}\right), \widehat{{X}_{B}}={G}_{B}\left({X}_{A};{\theta }_{B}\right), s.t. d\left({X}_{B}, \widehat{{X}_{B}}\right)\le \epsilon$$

24 $$\widehat{{X}_{B}}={G}_{B}\left({X}_{A};{\theta }_{B}\right), \widehat{{X}_{A}}={G}_{A}\left({X}_{B};{\theta }_{A}\right), s.t. d\left({X}_{A}, \widehat{{X}_{A}}\right)\le \epsilon$$

where *d* (·, ·) = distance metric between the input image and the reconstructed image (cycle-consistency constraint), and both *θ*_*A*_ and *θ*_*H*_ are the model parameters. StainGAN is trained to minimize adversarial and cycle-consistency loss (see Algorithm *5* for StainGAN training details). The cycle-consistency loss ensures that the output from *G*_A_ can be reconstructed back to the input for *G*_*B*_, and similarly, the output from *G*_*B*_ can be reconstructed back to the input for *G*_A_. The adversarial loss assures that the stain of the reconstructed images is coherent with the actual stain distribution.

Where the cycle-consistency loss for the $B\to A\to B$ cycle*,* ${\mathcal{L}}_{cycle}^{(B\to A\to B)}$ is described as follow:

25 $${\mathcal{L}}_{cycle}^{(B\to A\to B)}=\frac{1}{m}\sum_{i=1}^{m}{({{b}^{(i)}-D}_{A\to B}\left({G}_{B\to A}({b}^{\left(i\right)})\right))}^{2}$$

**Algorithm 5:**
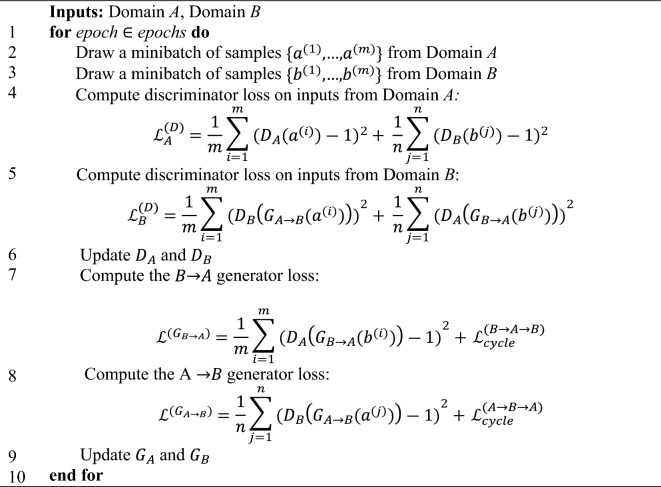
StainGAN Training Loop

#### StainNet

StainNet^47^ normalizes the source dataset by learning the color mapping relationship from the target dataset and adjusting its color value pixel by pixel. StainNet is a CNN comprising three convolutional layers with 32 kernels. StainNet necessitates the pairing of source and target images to facilitate the learning of color space conversion from the source to the target. Therefore, StainNet relies on the output of StainGAN to obtain the paired images. Specifically, we treat StainGAN as the teacher model while StainNet as the student model. The output images from StainGAN are treated as truth labels for the StainNet to train. Thus, the primary objective of the StainNet is to minimize the *L1* loss with *SGD* optimizer corresponding to the normalized images generated by StainGAN (see Algorithm *6* for StainNet training details). The mapping association of StainGAN is contingent on the image content. Therefore, by training on images normalized by StainGAN, StainNet can convert the content-based mapping association of StainGAN into a pixel value-based mapping.

**Algorithm 6:**
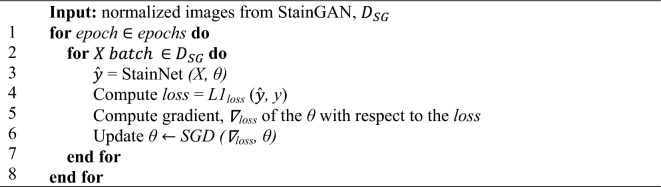
StainNet Training Loop

### Implementation details

This section outlines the implementation details of training CNN models on various stain-normalized datasets. The objective is to evaluate the performance of these models when trained on diverse stain-normalized datasets.

#### Dataset description

##### FBCG dataset

We adopted the dataset strategy proposed by Abdelli et al.^19^, known as the Four Breast Cancer Grades (FBCG) dataset to address the limitations of the existing small IDC grading datasets. The FBCG dataset entails 888 RGB H&E stained 400X-magnification IDC histopathological images with four classes: Grade 0 (G0), Grade 1 (G1), Grade 2 (G2), and Grade 3 (G3). The images in the G0 class (588 in total) are sourced from the Benign class of the BreaKHis dataset^63^, captured at a 400X magnification. The images in the other classes (300 in total) are sourced from the BCHI dataset^69^. Table 1 summarizes the composition of the FBCG dataset.

**Table 1:** The class distribution and proposed train-test split of the FBCG dataset.

		Grade 0	Grade 1	Grade 2	Grade 3	Total
FBCG Dataset	Train set	470	86	82	73	711
	Test set	118	21	20	18	177
	Total	588	107	102	91	888

##### BCHI dataset

**Figure 3:**
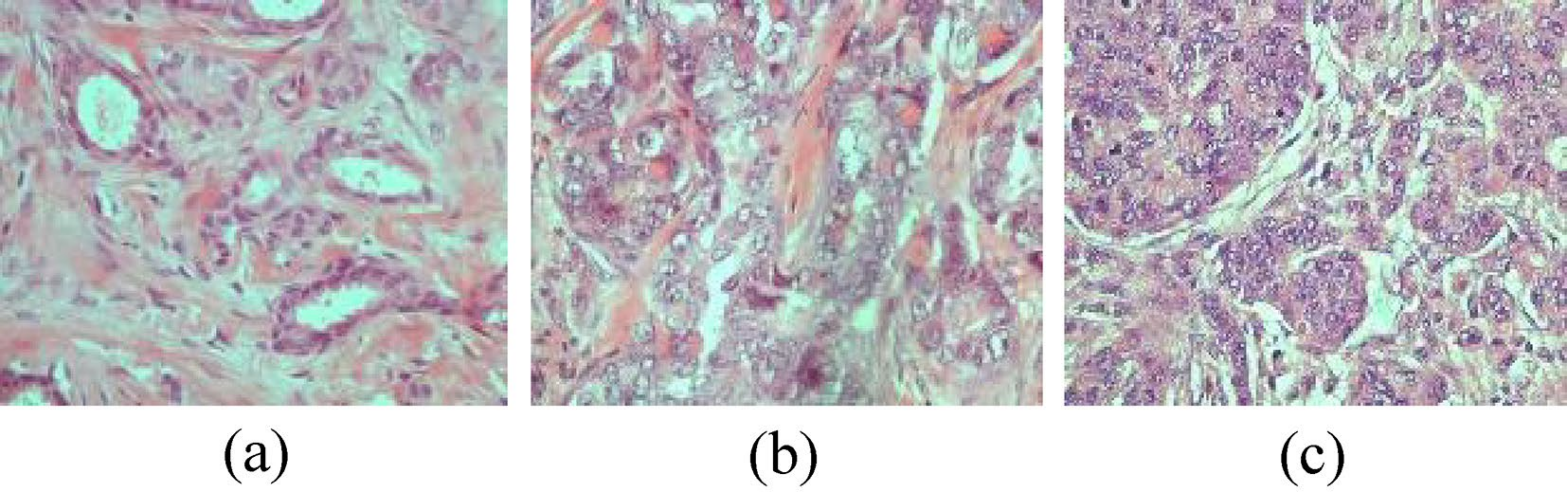
Samples images with 400X magnification from the BCHI dataset: (**a**) Grade 1, (**b**) Grade 2, (**c**) Grade 3.

The Breast Carcinoma Histological Images (BCHI) dataset^69^ includes 300 H&E-stained breast histopathology images (1280 × 960 pixels) from the pathology department at "Agios Pavlos" Hospital in Thessaloniki, Greece. The images, which depict carcinoma specimens, are categorized into three grades: Grade 1 (with 107 images), Grade 2 (with 102 images), and Grade 3 (with 91 images). These images are sourced from 21 IDC patients. The images were captured using a Nikon camera and a 40X magnification objective lens on a compound microscope (see Fig. 3).

##### BreaKHis dataset

**Figure 4:**
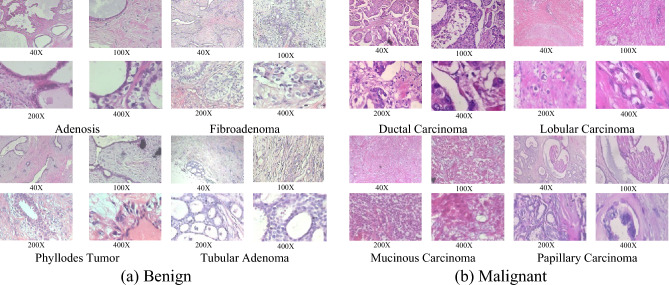
Samples from the BreaKHis dataset distributed into two major classes: (**a**) Benign and (**b**) Malignant with four magnification factors.

The BreaKHis dataset^63^ comprises 7909 histopathological images of breast cancer, sourced from 82 patients. Initially, the H&E-stained slide was captured at four magnification factors (40X, 100X, 200X, and 400X), using four objective lenses (4X, 10X, 20X, and 40X). These images were then converted into digital RGB format dimensions of 700 by 460 pixels. The BreaKHis is primarily divided into two categories: (1) Benign (2480 images) and (2) Malignant (5429 images). Each of the category can be further subdivided into four subclasses. For the Benign class, these are: (1) Adenosis, (2) Fibroadenoma, (3) Phyllodes Tumor, and (4) Tubular Adenoma. For the Malignant class, the subclasses are: (1) Ductal Carcinoma, (2) Lobular Carcinoma, (3) Mucinous Carcinoma, and (4) Papillary Carcinoma (see Fig. 4). Table 2 provides a detailed distribution of the images by major classes and magnifications within the BreaKHis dataset.

**Table 2:** The BreaKHis image distribution by two major classes and four magnifications.

Magnification	Benign	Malignant	Total
40x	625	1,370	1,995
100x	644	1,437	2,081
200x	623	1,390	2,013
400x	588	1,232	1,820
Total	2,480	5,429	7,909

#### Experiment setup

In this study, we assessed the base dataset (original FBCG dataset), represented as ${D}_{B}$, comprising *2D* pixel elements with three *RGB* channels and their corresponding ground truth labels. We employed six selected SN techniques: Reinhard (*R)*, Macenko (*M*), SPCN *(S)*, ACD *(A)*, StainGAN (*SG*) and StainNet (*ST*) on ${D}_{B}$ to create stain-normalized dataset ${D}_{SN,T}$. Here, $SN\in \{R, M, S, A, SG, ST\}$ denotes the SN technique and $T\in \{T1, T2, T3, T4, T5, \varnothing \}$ (The $\varnothing$ is reserved for *SG* and *ST* where *T* is not required) signifies the template used. For example, ${D}_{R,T1}$ refers to the dataset normalized using the Reinhard technique with Template *T1*. Each dataset was split into a training set ${D}_{TR}$ and a test set ${D}_{TS}$ in an 80%-20% ratio (see Table 1 for the train test split).

We conducted Stratified Five-fold Cross-validation (SFFCV) on the training set ${D}_{TR}$ by dividing it into five subsets, using one subset for validation and the remaining subsets for training. With SFFCV, we can compute the mean *μ* and standard deviation *σ* from results obtained from each subset for model stability evaluation (based on *σ*) and hyperparameters optimization. This process helps to minimize result variability, promote model stability, and provide a comprehensive performance evaluation across the base dataset ${D}_{B}$. After SFFCV, we retrained our models with the whole training set ${D}_{TR}$ and tested on the testing set ${D}_{TS}$ to obtain our baseline test result. Then, we repeated this procedure the stain-normalized training sets ${D}_{TR}\in {D}_{SN, T}$ and tested on the stain-normalized testing sets ${D}_{TS}\in {D}_{SN, T}$ to investigate the performance of CNN models trained with different stain-normalized datasets (see Algorithm *8*).

Before model training, we generated batches of pre-processed image data from each dataset with different image pre-processing functions (see Table 5). We also applied the class-weighting algorithm to address imbalanced classes in each dataset, ensuring the model converges for the minor classes in minimizing loss^70^. Equation (26) below describes the class-weighting algorithm.

26 $$Class Weight= \frac{N}{{N}_{c}\times {N}_{sc}}$$

where $N$ = number of images of all classes, ${N}_{c}$ = number of classes and ${N}_{sc}$ = number of images per class.

**Figure 5:**
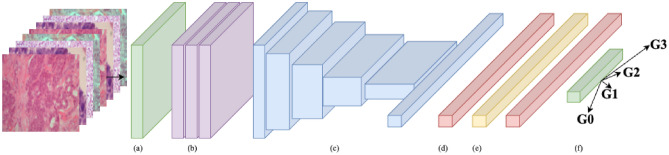
The structure of the model: (**a**) input layer, (**b**) augmentation layers, (**c**) feature extractor (non-trainable), (**d**) dropout layer, (**e**) dense layer (trainable), and (**f**) output prediction layer (trainable).

For the model implementation, we adhered to the approach outlined in Voon et al.^56^. We utilized seven pre-trained CNNs (see Table 3) from ImageNet^71^ and ImageNet-21k^72^ as feature extractors. Each model is composed of an input layer, augmentation layers, a feature *extractor* denoted as *f*_*θ*_ with model parameter *θ*, and a classifier denoted as $C\left(\cdot \right|W)$ with weight matrix ${W}\in {\mathbb{R}}^{d\times c}$. Our model structure is illustrated in Fig. 5. The classifier $C\left(\cdot \right|W)$ includes of two dropout layers and dense layers, with the final dense layer equipped with four neurons and a SoftMax activation function for classification (see Table 4). We kept the parameter *θ* in the *f*_*θ*_ fixed and trained a new classifier $C\left(\cdot \right|W)$ on each training set ${D}_{TR}$ by minimizing the weighted categorical cross-entropy loss, ${WCCE}_{loss}$ (see Eq. (27)) using the Adam Optimizer^73^. Subsequently, we tested each trained classifier on its corresponding testing set ${D}_{TS}$*.* The optimal learning rate and the number of epochs for model training were determined through SFFCV (see Table 5).

**Table 3:** Description of the seven pre-trained CNNs in terms of their characteristics, number of FLOPs, and number of parameters.

Architecture	Characteristic	FLOPs (B)	Parameters (M)
EfficientNet-B0 (EB0)^48^	Compound scaling	0.39	5.3
EfficientNet-V2-B0(EB0V2)^49^	Progressive learning	0.72	7.1
EfficientNet-V2-B0-21k (EB0V2-21k)^49^	Progressive learning	0.72	7.1
ResNet-V1-50 (RN1)^50^	Residual learning	4.1	25.6
ResNet-V2-50 (RN2)^51^	Identity mapping	4.1	25.6
MobileNet-V1 (MB1)^52^	Depth-wise separable convolutions	0.6	4.2
MobileNet-V2 (MB2)^53^	Inverted residuals and linear bottlenecks	0.3	3.4

**Table 4:** The structure of the model which follows the implementation of Voon et al.^56^.

Block	Detail
0	Input layer, shape = (224, 224, 3)
1	Augmentation layers:
	Random flip layer, mode = horizontal and vertical
	Random rotation layer, factor = 0.2
	Random zoom layer, height factor = 0.2
2	Feature extractor *f*_*θ*_
3	Dropout layer, rate = 0.5
	Dense layer, 256 neurons with ReLU function
	Dropout layer, rate = 0.4
	Dense layer, 4 neurons with SoftMax function for final prediction

**Table 5:** Details of image pre-processing and hyperparameters for model compilation.

	Operation	Value
Pre-processing function	Rescale	1./255
	Resize	224 by 224 pixels
	Shuffle	true
	Seed	123
	Batch	16
Hyperparameter	Loss function	${WCCE}_{loss}$
	Optimizer	Adam
	Learning rate	0.001
	Metric	accuracy
	Epochs	100

27 $${WCCE}_{loss}= -{w}_{j}*log\left(\frac{{e}^{{s}_{p}}}{{\sum }_{j}^{c}{e}^{{s}_{j}}}\right)$$

where ${w}_{j}$ = classes weights, ${S}_{p}=$ positive output score and ${S}_{j}=$ other classes output scores.

We primarily utilized the Balanced Accuracy (BAC) score as the evaluation metric for assessing model performance. The BAC, which calculates the average recall of each class, is computed using true positives (TP), true negatives (TN), false positives (FP), and false negatives (FN). The following mathematical expression defines the BAC:

28 $$BAC= \frac{1}{|{N}_{c}|}{\sum }_{i=1}^{|{N}_{c}|}\frac{{TP}_{i}}{{TP}_{i}+{FN}_{i}}$$

**Algorithm 7:**
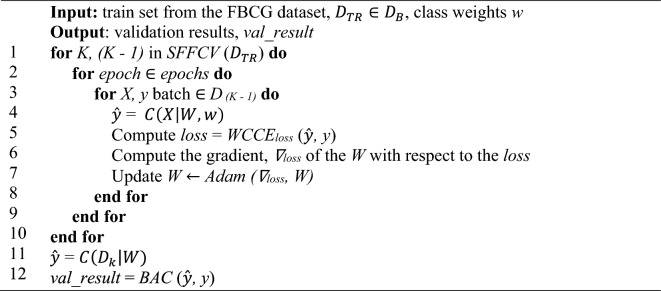
SFFCV Model Training and Validation Loop

**Algorithm 8:**
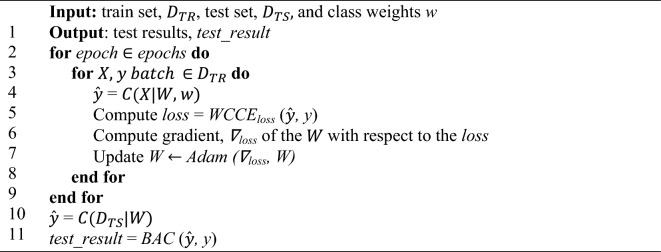
Model Training and Test Loop

## Results and discussion

### Results of stratified five-fold cross-validation

Table 6 presents the cross-validation and test outcomes of the seven models trained on the base dataset ${D}_{B}$. Please note, the test result forms the baseline for subsequent comparisons. Interestingly, all models secured high BAC scores (> 0.9) in the base test set ${D}_{TS}\in {D}_{B}$. Among all models, the EB0V2-21k and MB1 models achieve the highest BAC score (0.9524). For the validation result, we observe that the EB0V2-21k model achieves the highest BAC with relatively high stability (*μ* = 0.9666, *σ* = 0.0185). Generally, all models show low result variability. In other words, the models can generalize well across different subsets in ${D}_{TR}$.

**Table 6:** Cross-Validation and test BACs of seven models trained in ${D}_{B}$.The bolded values represent the highest score in each section.

Model	SFFCV (*μ* ± *σ*)	Test
EB0	0.9303 ± 0.0322	0.9518
EB0V2	0.9076 ± 0.0398	0.9024
EB0V2-21k	**0.9666 ± 0.0185**	**0.9524**
RN1	0.9253 ± 0.0310	0.9239
RN2	0.9346 ± 0.0156	0.9198
MB1	0.9518 ± 0.0232	**0.9524**
MB2	0.9362 ± 0.0322	0.9128
*μ* ± *σ*	**0.9361 ± 0.0189**	0.9308 ± 0.0211

### Results of conventional stain normalization techniques

**Figure 6:**
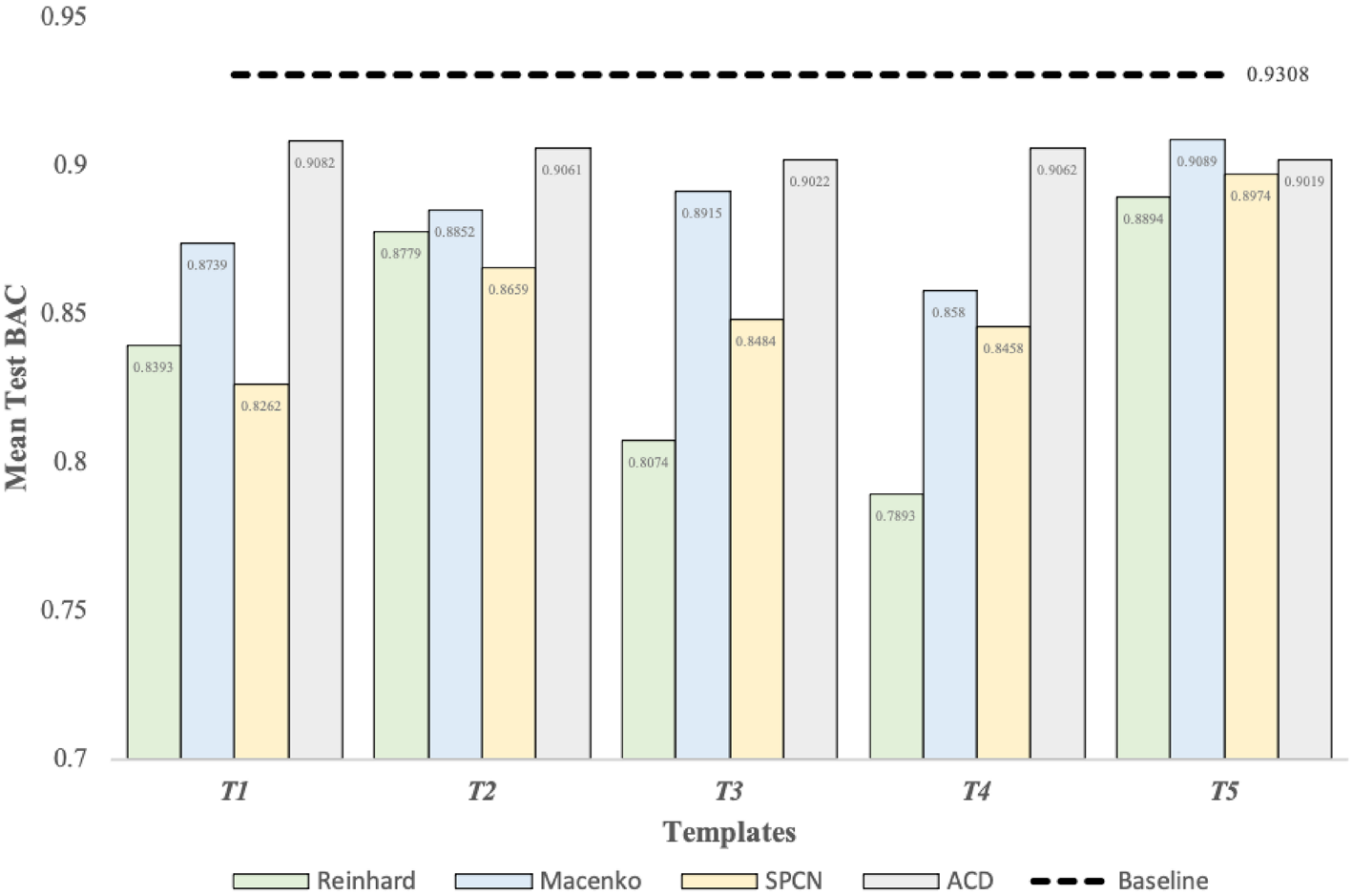
The mean test BAC scores of the seven models across *T* with different conventional SN techniques from Supplementary Tables 1, 2, 3, and 4. The ACD technique tops other techniques across all templates but failed to outperform the baseline result.

Figure 6, derived from Supplementary Tables 2–5, depicts the mean test BAC scores of seven models trained with datasets normalized using Reinhard, Macenko, SPCN, and ACD techniques across *T*. Our results underscore that the ACD technique yielded the highest average BAC score (0.905) across *T*, succeeded by Macenko (0.8835), SPCN (0.8567), and Reinhard (0.8407) techniques. Nonetheless, none of the techniques managed to surpass the baseline result (0.9308). Among *T*, *T*5 yields the highest average BAC scores with Reinhard, Macenko, and SPCN techniques, whereas *T*1 attains the highest BAC using the ACD technique. *T*5 consistently achieves good results across different SN techniques. The superior performance of *T*5 may be attributed to the consideration of the dominant color in the target images. In histopathological images, the dominant color often corresponds to the stain used, which carries crucial information for classification tasks. By effectively capturing the dominant color, *T*5 can guide the SN process to better preserve or standardize this critical information, leading to improved classification performance.

Among conventional SN techniques, we noted that template selection minimally impacts the ACD technique due to its small σ (refer to Supplementary Table 4). In contrast, the Reinhard, Macenko and SPCN techniques are more heavily affected by the template selection. Hence, we propose that judicious template selection is crucial for Reinhard, Macenko, and SPCN techniques. Additionally, we suggest using the ACD technique for SN over other techniques if a conventional SN technique is required in the image pre-processing pipeline.

### Results of deep learning-based stain normalization techniques

**Figure 7:**
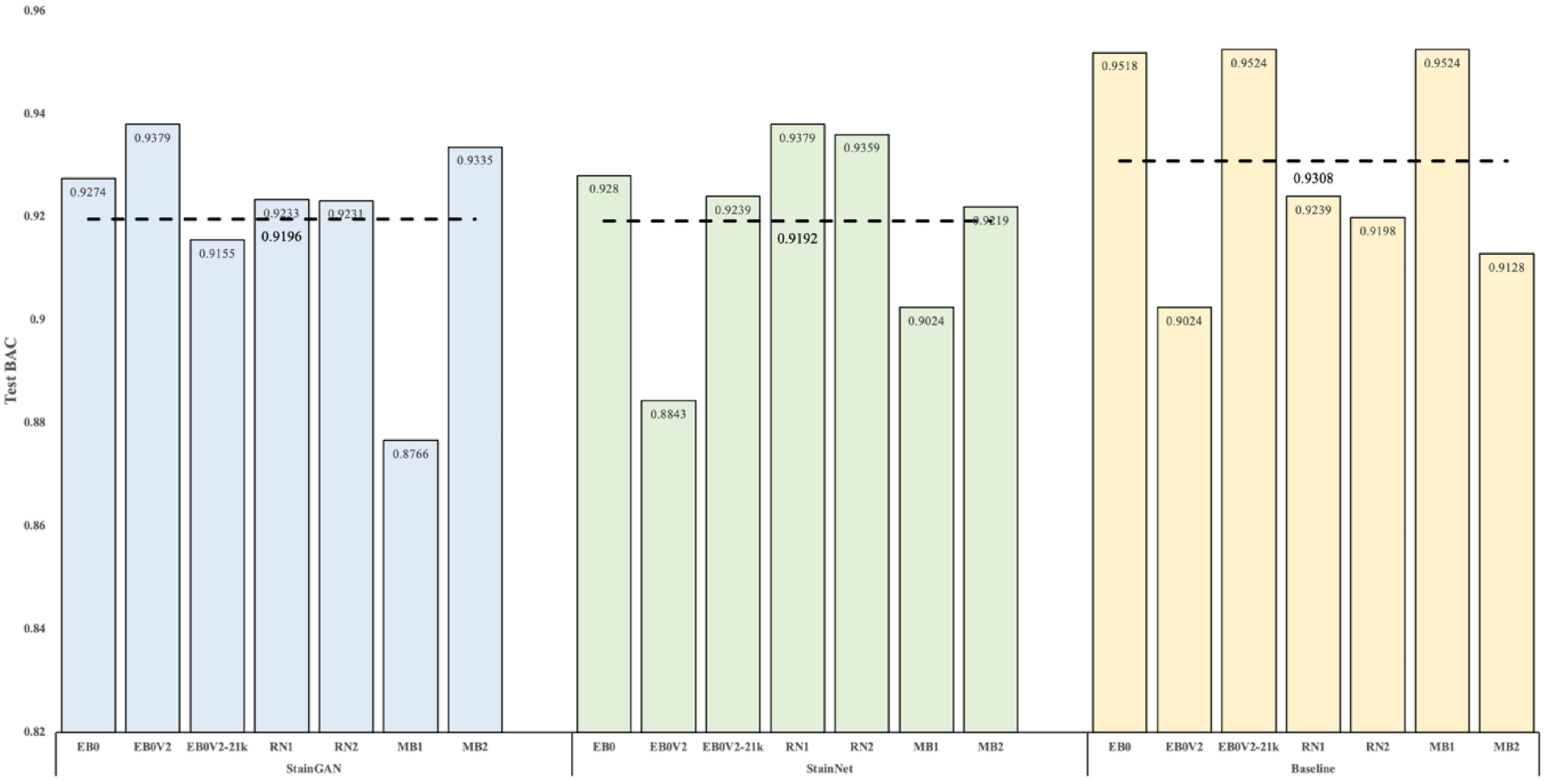
The test BAC scores of seven models trained with StainGAN-normalized, StainNet-normalized, and non-normalized datasets. Although the results are comparable among the deep learning-based SN techniques, the mean BAC scores of the seven models trained in the StainGAN-normalized dataset achieve slightly higher than models trained in the StainNet-normalized dataset but lower than the baseline result.

Figure 7, derived from Table 6 and Supplementary Table 5, depicts the test BAC scores of seven models trained with StainGAN-normalized, StainNet-normalized, and non-normalized datasets. We noted a high similarity in the performance of models trained with StainGAN-normalized and StainNet-normalized datasets, aligning with the findings by Kang et al.^47^. Nonetheless, models trained with the StainGAN-normalized dataset exhibited marginally higher mean test BAC scores (0.9196) than those trained with the StainNet-normalized dataset (0.9192). Additionally, our findings highlight that deep learning-based SN techniques failed to outperform the baseline result. Therefore, our results underscore the importance of context-specific application of these techniques and suggests that they may not universally lead to improved performance in every scenario.

### Evaluation of the effectiveness of stain normalization in the idc grading task

**Figure 8:**
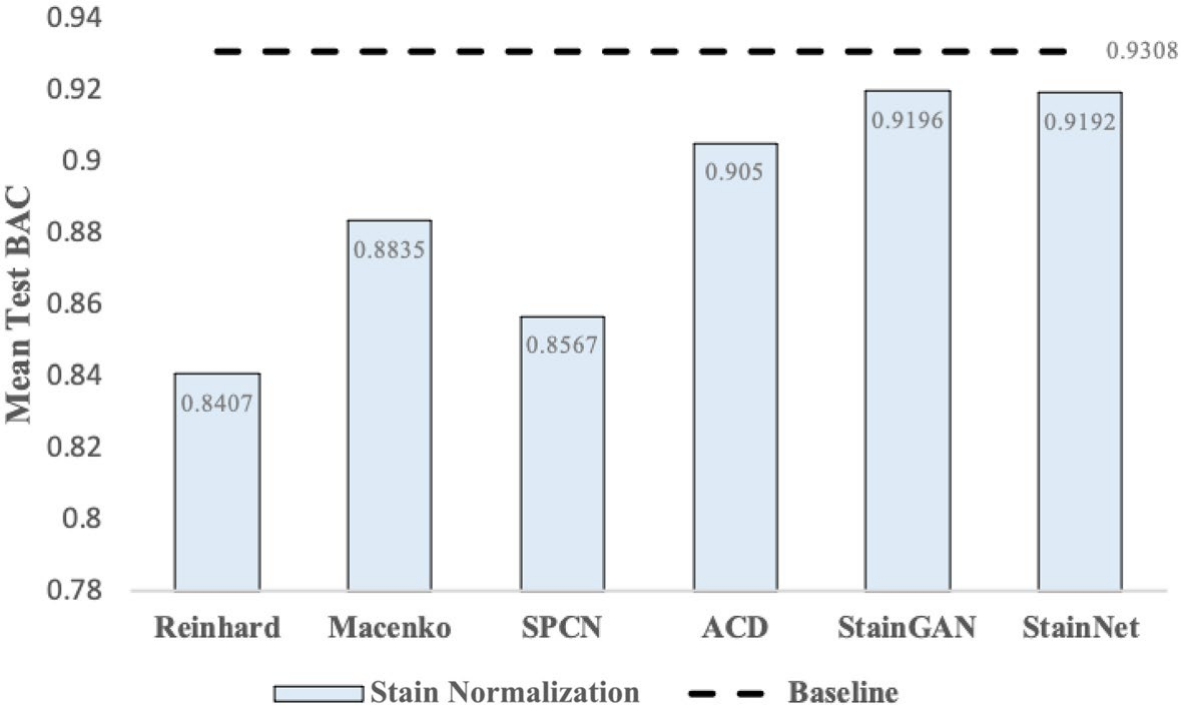
The mean test BAC scores of the seven models trained in six different stain-normalized and the non-normalized FBCG datasets. Among the six SN techniques, the StainGAN technique outperforms other SN techniques. However, the baseline result tops the best SN results by 0.0112 score.

In this section, we assessed the efficacy of SN in IDC grading using the FBCG dataset. Figure 8 illustrates the mean test BAC scores of the seven models trained in six different stain-normalized and the non-normalized datasets. Our results underscore that models trained with StainGAN-normalized images surpass those trained with other stain-normalized images. Hence, we compared the test mean BAC score between models trained with the StainGAN-normalized dataset and models trained with the non-normalized dataset. The results of the *t*-test indicated that the mean BAC score was statistically insignificant between models trained with the StainGAN-normalized FBCG dataset (*μ* = 0.9196, *σ* = 0.0188) and models trained with the non-normalized dataset (*μ* = 0.9308, *σ* = 0.0211), *p* = 0.11. The *p*-value indicates that the probability of obtaining the results is 11% by chance. Since the *p*-value of 0.11, higher than the significance level, *α* = 0.05, suggests the difference in mean BAC scores between models trained with the StainGAN-normalized dataset and models trained with the non-normalized dataset is statistically insignificant. Consequently, we did not dismiss the null hypothesis, suggesting no significant difference in the performance of stain-normalized and non-normalized datasets for IDC grading tasks.

Furthermore, it is possible that SN techniques strip distinct color features^67^ from IDC images, leading to poorer model performance. Our findings oppose the presumption that SN is essential to accomplish good performance in histopathological classification tasks, aligning with other similar studies^26–30^. Therefore, we suggest that future studies should conduct ablation studies with the employed dataset regarding the effectiveness of SN in their applications. Despite the ineffectiveness of SN in our IDC grading task, we acknowledge its contribution as evidenced by its benefits in other studies^31–35^. In response to the claim that SN may eliminate color features in IDC images, future studies could explore the influence of these color features on the generalizability of the CNN.

In summary, the impact of SN on recent breast cancer histopathological studies has been the subject of debate. Our study aimed to elucidate this matter by scrutinizing the efficacy of SN techniques in breast cancer histopathological classification tasks, particularly in IDC grading, using CNNs. We selected six conventional and deep learning-based SN techniques to evaluate their effectiveness, along with seven pre-trained CNNs from ImageNet and ImageNet-21k as feature extractors. Our findings revealed that the impact of SN on this task was not statistically significant. Consequently, we did not reject the null hypothesis, suggesting that there was no substantial difference in effectiveness between stain-normalized and non-normalized datasets for IDC grading tasks. This outcome challenges the prevailing assumption that SN invariably enhances classification outcomes, thereby contributing a nuanced perspective to the discourse on the role of SN in breast cancer histopathological studies.

### Limitations of study

The scope and limitations of our study focused to investigating the effectiveness of SN on IDC grading using only the FBCG dataset. Future work will incorporate other IDC grading datasets, such as DataBiox^74^ and PathoIDCG^55^. Additionally, our study did not account for potential variations in staining protocols across different centers. This is a significant consideration, since the staining process can greatly influence the color and intensity of histopathological images, which in turn can impact the performance of the model. While our findings underscore the impact of SN on IDC grading, they may not extend to scenarios where training and testing data come from separate centers. This limitation will be addressed in future work.

We utilized six different SN techniques in this study and plan to incorporate additional techniques^39,45,61,75,76^ in future research. Subsequently, we selected five templates from the PCam train set to accommodate the Camelyon16 pre-trained StainGAN and StainNet. These templates were chosen as the results of applying three different similarity functions: (1) Cosine Similarity (${SIM}_{C}$), (2) Mean Square Error (MSE), and (3) the Structural Similarity Index (SSIM), along with considering the most dominant color of the average image and the target images. The selection process aimed to identify templates that closely resemble the stain distributions in the target dataset. By using different similarity metrics, we were able to ensure that each template provided a unique perspective on the target data. Nonetheless, the five templates selected may not fully represent the color characteristics of the target dataset. This selection process has an empirical aspect, as there is no one-size-fits-all rule for template selection in style transfer.

For the model implementation, we only selected seven pre-trained CNNs for evaluations based on the implementation of Voon et al.^56^. We omitted other state-of-the-art CNNs^77–79^ from our study but reserved them for future work. This study focused on the effectiveness of SN in the application; thus, we disregarded advanced model optimizations such as model fine-tuning and hyperparameter tuning.

### Challenges of study

We encountered two significant challenges during the experimentation: (1) data imbalance and (2) model overfitting. An imbalanced dataset may inject bias into the CNN, causing the CNN to favor the majority class. Hence, we implemented the class-weighting algorithm that assigned higher weights to minority classes to increase the penalty. Given the relatively small size of our FBCG dataset compared to other breast cancer-related datasets, we noted a risk of model overfitting with complex CNN architectures. To mitigate this, we incorporated augmentation layers into our model for enhanced data diversity and added two dropout layers in our classifier to randomly nullify input units, thereby preventing overfitting during training.

## Conclusion

In this study, we set out to address the question of the effectiveness of Stain Normalization (SN) in the task of Invasive Ductal Carcinoma (IDC) grading. To accomplish this, we utilized seven pre-trained Convolutional Neural Network (CNN) models as feature extractors to classify the FBCG dataset into four IDC grades. The FBCG dataset was stain-normalized using six techniques: Reinhard, Macenko, SPCN, ACD, StainGAN, and StainNet. For the conventional SN techniques, we selected five templates to investigate their impacts on each method. We conducted a comparative analysis of models trained with and without SN to understand the impact of SN on the classification results. Our findings revealed a *p*-value of 0.11 when comparing the test mean Balanced Accuracy (BAC) score of models trained with StainGAN-normalized (best-performing SN technique) images and non-normalized images. This indicates that there is no statistically significant difference in the effectiveness of stain-normalized and non-normalized datasets for IDC grading tasks. Contrary to common belief, our study suggests that SN may not be as crucial for histopathological classification tasks as previously thought. However, if SN is required in the image pre-processing pipeline, we recommend StainGAN, StainNet, and ACD techniques due to their relative performance in stain-normalizing images. Looking forward, in addition to extending our future work with the consideration mentioned in Sect. 4.5, we plan to examine the generalizability of the CNN model with respect to color features in IDC. Additionally, we aim to explore the inconsistent effects of SN on different breast cancer histopathological classification tasks.

### Supplementary Information

Supplementary Table 1.

Supplementary Table 2.

Supplementary Table 3.

Supplementary Table 4.

Supplementary Table 5.

## Data Availability

The origin datasets combined for the current study are available in the Four Breast Cancer Grades (FBCG) Dataset, https://web.inf.ufpr.br/vri/databases/breast-cancer-histopathological-database-breakhis/, and breast carcinoma histological images from the Department of Pathology, https://zenodo.org/record/834910#.WXhxt4jrPcs. Should there be any inquiries regarding the employed datasets, please contact the corresponding author, Dr. Hum Yan Chai (humyc@utar.edu.my) for further information and clarification.
