# Supplementary material for: Evaluating the effectiveness of stain normalization techniques in automated grading of invasive ductal carcinoma histopathological images
Source: Sci Rep. 2023 Nov 22;13:20518. doi: 10.1038/s41598-023-46619-6 (PMC10665422; doi:10.1038/s41598-023-46619-6)
Supplement: Supplementary file 1 — Supplementary Table 1. [file 41598_2023_46619_MOESM1_ESM.pdf]

Supplementary Table 1. Test BACs of CNNs trained with $D_{R,\ T}$. The bolded values represent the highest score in each section.

Model	T1	T2	T3	T4	T5	$\mu \pm \sigma$
**EB0**	0.8843	0.8565	0.8212	0.7855	0.9114	
**EB0V2**	0.6656	0.8626	0.5975	0.6209	0.8653	
**EB0V2-21k**	0.8425	0.8739	0.7941	0.7362	**0.8975**	
**RN1**	**0.8969**	0.9115	**0.8698**	**0.8817**	0.8891	
**RN2**	0.8571	**0.9213**	0.8585	0.8485	0.8969	
**MB1**	0.8620	0.8353	0.8569	0.8363	0.9155	
**MB2**	0.8669	0.8844	0.8541	0.8157	0.8498	
**Average**	0.8393	0.8779	0.8074	0.7893	**0.8894**	0.8407 ± 0.0388
