# Supplementary material for: Evaluating the effectiveness of stain normalization techniques in automated grading of invasive ductal carcinoma histopathological images
Source: Sci Rep. 2023 Nov 22;13:20518. doi: 10.1038/s41598-023-46619-6 (PMC10665422; doi:10.1038/s41598-023-46619-6)
Supplement: Supplementary file 2 — Supplementary Table 2. [file 41598_2023_46619_MOESM2_ESM.pdf]

Supplementary Table 2. Test BACs of CNNs trained with $D_{M, T}$. The bolded values represent the highest score in each section.

Model	T1	T2	T3	T4	T5	$\mu \pm \sigma$
**EB0**	0.8617	0.8472	0.8813	0.7991	0.9027	
**EB0V2**	0.8331	0.8664	0.8775	0.8363	0.8605	
**EB0V2-21k**	0.8817	0.9098	**0.9209**	0.8854	**0.9272**	
**RN1**	0.9036	**0.9161**	0.8736	**0.8856**	0.9204	
**RN2**	0.8332	0.8709	0.877	0.855	0.9042	
**MB1**	**0.9104**	0.903	0.9151	0.8778	0.9218	
**MB2**	0.8936	0.8827	0.8952	0.8671	0.9255	
**Average**	0.8739	0.8852	0.8915	0.8580	**0.9089**	0.8835 ± 0.0171
