# Supplementary material for: Evaluating the effectiveness of stain normalization techniques in automated grading of invasive ductal carcinoma histopathological images
Source: Sci Rep. 2023 Nov 22;13:20518. doi: 10.1038/s41598-023-46619-6 (PMC10665422; doi:10.1038/s41598-023-46619-6)
Supplement: Supplementary file 3 — Supplementary Table 3. [file 41598_2023_46619_MOESM3_ESM.pdf]

Supplementary Table 3. Test BACs of CNNs trained with $D_{S,T}$. The bolded values represent the highest score in each section.

Model	T1	T2	T3	T4	T5	$\mu \pm \sigma$
**EB0**	0.7945	**0.9073**	0.888	0.8686	0.9023	
**EB0V2**	0.7017	0.7791	0.7351	0.7482	0.8732	
**EB0V2-21k**	0.874	0.8866	0.8408	**0.9025**	0.8669	
**RN1**	**0.8954**	**0.9073**	**0.8891**	0.8582	0.9094	
**RN2**	0.8499	0.8802	0.8876	0.7904	**0.9352**	
**MB1**	0.8181	0.878	0.8272	0.902	0.8905	
**MB2**	0.8496	0.8227	0.871	0.8504	0.9046	
**Average**	0.8262	0.8659	0.8484	0.8458	**0.8974**	0.8567 ± 0.0239
