# Supplementary material for: Evaluating the effectiveness of stain normalization techniques in automated grading of invasive ductal carcinoma histopathological images
Source: Sci Rep. 2023 Nov 22;13:20518. doi: 10.1038/s41598-023-46619-6 (PMC10665422; doi:10.1038/s41598-023-46619-6)
Supplement: Supplementary file 4 — Supplementary Table 4. [file 41598_2023_46619_MOESM4_ESM.pdf]

Supplementary Table 4. Test BACs of CNNs trained with $D_{A, T}$. The bolded values represent the highest score in each section.

Model	T1	T2	T3	T4	T5	$\mu \pm \sigma$
**EB0**	0.928	**0.9483**	0.8877	0.9114	0.9189	
**EB0V2**	0.9003	0.8891	0.8454	0.876	0.812	
**EB0V2-21k**	**0.9378**	0.9254	0.922	**0.9379**	**0.9378**	
**RN1**	0.9198	0.8969	**0.9385**	0.9233	0.924	
**RN2**	0.8858	0.881	0.8976	0.8844	0.8984	
**MB1**	0.9155	0.903	0.9115	0.9117	0.9134	
**MB2**	0.8705	0.8987	0.9127	0.8984	0.9086	
**Average**	**0.9082**	0.9061	0.9022	0.9062	0.9019	0.905 ± 0.0025
