# Supplementary material for: Evaluating the effectiveness of stain normalization techniques in automated grading of invasive ductal carcinoma histopathological images
Source: Sci Rep. 2023 Nov 22;13:20518. doi: 10.1038/s41598-023-46619-6 (PMC10665422; doi:10.1038/s41598-023-46619-6)
Supplement: Supplementary file 5 — Supplementary Table 5. [file 41598_2023_46619_MOESM5_ESM.pdf]

Supplementary Table 5. BACs of CNNs trained with D_{SG} and D_{ST}. The bolded values represent the highest score in each section.

Model	StainGAN	StainNet
**EB0**	0.9274	0.928
**EB0V2**	**0.9379**	0.8843
**EB0V2-21k**	0.9155	0.9239
**RN1**	0.9233	**0.9379**
**RN2**	0.9231	0.9359
**MB1**	0.8766	0.9024
**MB2**	0.9335	0.9219
$\mu \pm \sigma$	**0.9196 ± 0.0188**	0.9192 ± 0.0179
